# Acute Heart Failure in Advanced Systemic Sclerosis With Multiorgan Involvement

**DOI:** 10.1155/cric/5532920

**Published:** 2026-07-21

**Authors:** Hanna Maria Eivindson, Esben Uggerby Naesser, Per Ivarsen, Brian Bridal Løgstrup

**Affiliations:** ^1^ Department of Cardiology, Aarhus University Hospital, Aarhus, Denmark, auh.dk; ^2^ Department of Rheumatology, Aarhus University Hospital, Aarhus, Denmark, auh.dk; ^3^ Department of Nephrology, Aarhus University Hospital, Aarhus, Denmark, auh.dk

## Abstract

**Background:**

Heart failure secondary to autoimmune diseases is uncommon. Systemic sclerosis (SSc) is a chronic autoimmune disorder marked by microvascular damage and progressive fibrosis in multiple organ systems.

**Case Summary:**

A man in his 60s, without cardiovascular risk factors, presented with worsening dyspnea (in cardiogenic pulmonary edema), chronic finger cyanosis with ischemic ulcers, progressive dysphagia for about 2 years, and a 10 kg weight loss over 6–12 months. Echocardiography showed a severely impaired left ventricular ejection fraction of 10%. Computer tomography excluded malignancy but demonstrated pleural effusion and pulmonary edema. The patient received immediate treatment for cardiogenic pulmonary edema and was stabilized. Further diagnostic evaluation confirmed diffuse cutaneous systemic sclerosis (dcSSc).

**Discussion:**

Primary cardiac involvement as an initial manifestation of dcSSc is rare and remains a significant source of morbidity and mortality.

**Take Home Messages**

A rapid interdisciplinary strategy for these complex cases is essential. Empirical management and absence of specific guidelines can delay timely, individualized therapy.

## 1. History of Presentation

**Figure 1:**
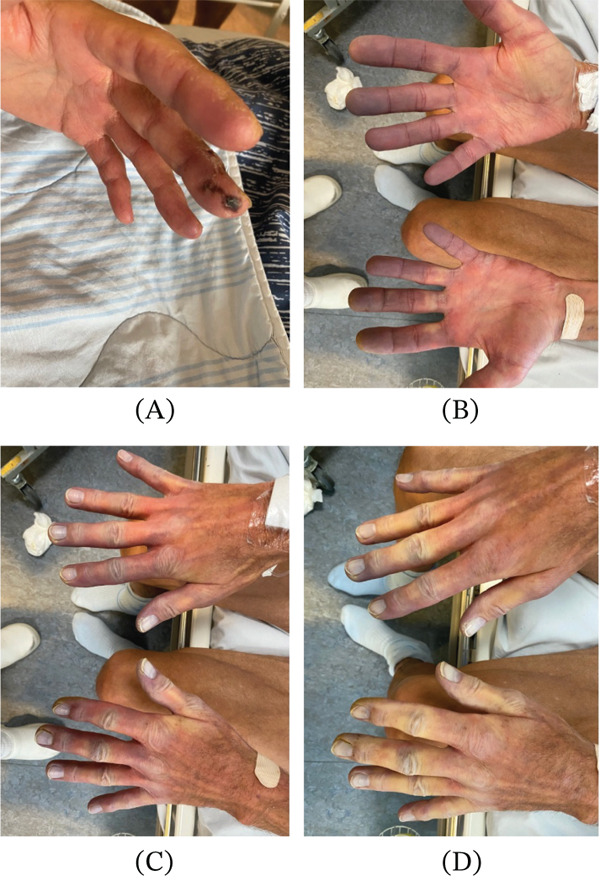
(A–D) Clinical objective signs of digital ischemia. Legend: sclerodactyly, cyanotic fingers (Raynaud′s phenomenon) with digital ulcerations located at the finger tips.

A man in his 60s presented to the community hospital′s emergency department with progressive dyspnea over a year, longstanding cyanosis of the fingers with ulcers (Figure 1), dysphagia for about 2 years, and a 10 kg weight loss over 6–12 months. The patient received no regular medications and had no smoking or alcohol abuse. Throughout the course of his symptoms, the patient did not seek medical attention until admission.

**Figure 2:**
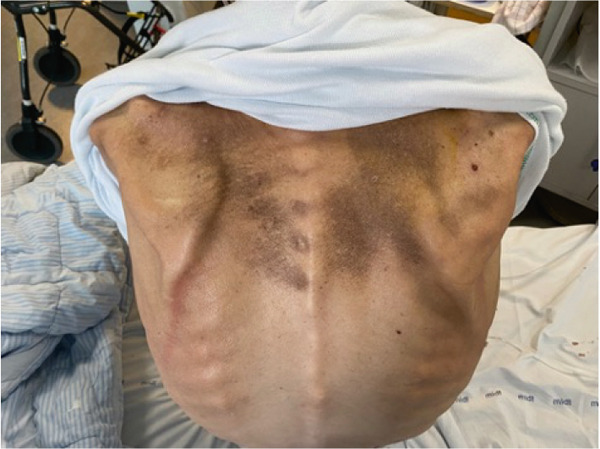
Clinical objective signs of the neck/back area. Legend: mottled pattern of hypopigmentation and hyperpigmentation (salt and pepper‐pigmentation) of the neck/back area.

On admission, the patient was cachectic (Figure 2), with tachycardia, dyspnea, and high blood pressure. Labs showed compensated metabolic acidosis with elevated lactate, renal failure, cardiac biomarkers suggestive of severe myocardial involvement, and mild inflammation. The patient presented as in cardiogenic pulmonary edema. Baseline clinical and paraclinical parameters are presented in Table 1.

**Table 1:** Baseline clinical and paraclinical parameters.

Vital parameters	Reference intervals	At admission
Respiratory rate per min	12–20	20
Peripheral oxygen saturation (%)	> 94	85
Blood pressure (mmHg)	90/60–120/80	144/101
Pulse (beats per min)	60–100	110
Temperature (Celsius)	36.5–37.5	36.0
Arterial blood test		
pH	7.37–7.45	7.40
pCO2 (kPa)	4.7–6.0	3.5
pO2 (kPa)	9.6–13.7	12.0
Oxygen saturation (%)	0.92–0.99	0.96
HCO3‐ bicarbonate (mmol/L)	22.5–26.9	19.0
Base excess (mmol/L)	(−2)–(+3)	−7
Lactate (mmol/L)	0.5–2.5	6.3
Venous blood test		
Troponin T	< 19 ng/L	699
NT pro‐BNP	< 300 ng/L	70,000
D‐dimer	< 0.70 mg/L FEU	4.0
Creatine kinase	40–280 U/L	590
Myoglobin	< 75 *μ*g/L	459
Lactate dehydrogenase (LDH)	105–205 U/L	615
Alanine transaminase (ALAT)	10–70 U/L	236
Bilirubin	5‐25 *μ*mol/L	35
International normalized ratio (INR)	< 1.2	1.4
Creatinine	60–105 *μ*mol/L	227
Estimated glomerular filtration rate (eGFR) (CKD‐EPI_crea_)	> 60 mL/min	25
P‐carbamide	3.5‐8.1 mmol/L	20.8
Potassium	3.6–4.6 mmol/L	4.8
Sodium	137–145 mmol/L	136
Calcium ion	1.18–1.32 mmol/L	1.07
Magnesium	0.70–1.10 mmol/L	1.00
C‐reactive protein (CRP)	< 8.0 mg/L	58
Leucocytes	3.50–10.0 × 10^9^/*L*	13.2
Neutrophils	2.0–7.0 × 10^9^/*L*	11.1
Hemoglobin	8.3–10.5 mmol/L	9.8

Electrocardiogram revealed atrial fibrillation with a frequency of 110 bpm. Computer tomography (CT) excluded malignancy and pulmonary embolism but showed pleural effusions, pulmonary edema, and cardiac ectasia. No interstitial lung disease was detected.

Echocardiography revealed a severely reduced left ventricular ejection fraction of 10% (no regional wall motion abnormalities were observed), pericardial effusion, right ventricular dilation, and reduced function (TAPSE 1.1 cm), and tricuspid valve regurgitation (Videos S1, S2, S3). The inferior vena cava was dilated, consistent with right‐sided heart failure (HF).

Given the diagnosis of acute HF, diuretics and vasodilators were initiated. Lactate levels normalized postdecongestion. Due to episodes of nonsustained ventricular tachycardia, amiodarone was started for arrhythmia, and the patient was transferred on Day 5 to a tertiary center.

After the transfer the patient was further stabilized. Given the patient′s history of cyanotic fingers, dysphagia, and weight loss, a rheumatology consultation was requested. The rheumatologist diagnosed the patient with diffuse cutaneous systemic sclerosis (dcSSc) based on clinical signs and positive autoantibodies (Table 2). The disease was considered long‐standing. Immunosuppressive treatment with prednisolone and cyclophosphamide was initiated. In addition to this treatment, a proton pump inhibitor was added. A gastric feeding tube was placed for nutritional support.

**Table 2:** Summary of clinical and biochemical findings.

Skin	Moderate thickness of the skin with a modified Rodnan skin score of 20 (out of 51) (18)	
	A carp‐like mouth	
	Facial telangiectasias and salt and pepper‐pigmentation of the neck area	
	Sclerodactyly, cyanotic fingers with digital ulcerations located at the findertips and Raynaud′s phenomenon capillaroscopy with dilated vessels	
Cardiopulmonary	Decompensated biventricular heart failure	
	Pleural fluid	
	No sign of interstitial lung disease	
Gastrointestinal	Malnourished/cachectic body with an BMI of 15 kg/m (Figure 1)	
	Severe dysphagia	
	No other GERD symptom nor gastrointestinal complains	
Urogenital	Kidney involvement with increased creatinine (maximum of 708 *μ*mol/L)	
	Mild proteinuria with an elevated urine‐albumin/creatinine‐ratio (312 mg/g)	
	No hypertension	
Mucous glands (sicca)	Dry mouth	
	No other mucosal dryness	
Autoantibodies	Positive	
	Antinuclear antibodies (ANA)	3.6 (< 1) ratio
		Positive (1:320, homogeneous pattern)
	Anti‐dsDNA	29 (10) 10^3^ IU/L
	Histon‐antibodies	7.5 (< 1.6) AU
	Anti‐PL7 IgG (antisynthetase)	Weak positive
	Rheumatoid factor IgM, CCP antibody, SCL70 antibody, fibrillarin antibody, RNA polymerase III antibodies, myeloperoxidase, anti‐Th/To antibodies, ribonuclease P/MRP POP1 antibody, anti‐RPC1 antibody, centromere B antibody, ribonecleoprotein antibody, proteinase 3 antibody, antinucleosome antibody.	

Treatment for the HF was considered; however, there was no room for the initiation of any of the four drug classes. The kidney function was severely impaired, so neither mineralocorticoid antagonists, renin–angiotensin‐receptor inhibition or SGLT‐2‐inhibition were initiated. Use of beta‐blockers at that time was considered inappropriate due to the acute HF situation and the presence of Raynaud′s phenomenon.

A persistently rising creatinine was attributed to hypoperfusion and dehydration rather than scleroderma renal crisis, which can be associated with dcSSc. Renal crisis was considered unlikely due to the absence of hypertension, hemolysis, thrombocytopenia, and the negative RNA polymerase III antibodies [1]. The patient exhibited acceptable diuresis, thus nonoliguric renal failure. Hemodialysis was started due to severe uremia (Day 7). Urea was considered a more reliable indicator of renal status due to the low muscle mass.

Due to the increased risk of pulmonal hypertension in patients with dcSSc [2] a right heart catheterization was performed (Table 3). It revealed normal pulmonary pressures and normal filling pressures, but a markedly low cardiac index, prompting dobutamine therapy (Day 10) to improve renal perfusion and cardiac output. The hemodynamics stabilized with a blood pressure of 112/79 mmHg.

**Table 3:** Right heart catheterization by thermodilution method.

	References	Results
Right atrial pressure (mmHg)	1–5	6
Right ventricle pressure (mmHg) (systolic/diastolic)	15–30/1–7	29/0
Pulmonary artery pressure (mmHg) (systolic/diastolic/mean)	15–30/4–12/9–19	29/? (nonvalid curve)
Pulmonary capillary wedge pressure (mmHg)	4–12	5
Cardiac output (L/min)	4.0–6.0	1.8
Cardiac index (L/min/m^2^)	> 2.5	1.1
Venous oxygen saturation (%)	60–80	67
Arterial oxygen saturation (%)	95–100	99

## 2. Past Medical History

The patient had a history of appendicitis and recurrent erysipelas. He had no family history of autoimmune or cardiovascular diseases.

## 3. Follow‐Up and Outcome

During follow‐up echocardiography (Day 11) showed left ventricular ejection fraction improvement to 35%–40% during dobutamine infusion (Videos S4 and S5). Right‐sided dilation persisted. No pulmonary hypertension was observed.

Cardiac magnetic resonance imaging (MRI) (without contrast due to renal dysfunction) revealed myocardial edema, reduced ejection fraction (44%) and reduced right ventricular ejection fraction (25%), with no ischemic signs (Day 16). The MRI exam suggested myocarditis. High‐dose corticosteroids were initiated in addition to proton pump inhibitor.

Due to multiorgan failure, the patient was non eligible for mechanical circulatory support or heart transplantation.

In the fourth week of hospitalization, the patient developed melena and anemia (Days 22–31). Gastroscopy confirmed a bleeding duodenal ulcer, treated with endoscopic clipping. A subsequent CT scan indicated pneumoperitoneum, likely from micro perforation of the ulcer. Conservative management was pursued due to comorbidities. Despite stabilization efforts, clinical deterioration followed. Blood cultures identified *Serratia marcescens*, indicating gastrointestinal sepsis. The patient later developed apnea with desaturation and passed away.

## 4. Discussion

This case highlights dcSSc with multiorgan involvement, presenting primarily as acute decompensated HF. Though cardiac complications occur in 15%–35% of dcSSc patients [2, 3], the actual prevalence is likely underreported due to variable diagnostic criteria [4]. Regardless, HF secondary to dcSSc remains rare due to the overall low incidence of the disease. Systemic sclerosis is a rare autoimmune connective tissue disease with an estimated incidence of 10–25 cases per million person‐years and a prevalence of approximately 100–300 cases per million inhabitants, although geographic variation exists. The disease most commonly presents between 40 and 60 years of age and shows a marked female predominance with female‐to‐male ratios ranging from 4:1 to 6:1. dcSSc accounts for approximately 20%–30% of cases and is generally associated with a more aggressive clinical course and higher risk of internal organ involvement. Therefore, the present case is notable both because of the patient′s sex and because severe cardiac involvement was among the dominant presenting manifestations [5–7].

At presentation, the patient exhibited severe left ventricular systolic dysfunction in the absence of alternative common etiologies such as hypertension, valvular heart disease, alcohol misuse, or exposure to cardiotoxic agents. Cardiac MRI did not demonstrate a pattern consistent with ischemic cardiomyopathy, supporting a nonischemic process in the setting of systemic autoimmune disease rather than a definitive dcSSc‐specific cardiac mechanism.

However, given the presence of anti‐PL‐7 antibodies and MRI findings suggestive of myocarditis, the differential diagnosis was broadened to include inflammatory and overlap connective tissue disease processes.

Autoimmune serology demonstrated ANA positivity at a titer of 1:320 with a homogeneous indirect immunofluorescence (IIF) pattern. This pattern is nonspecific and may be observed across several systemic autoimmune rheumatic diseases and therefore requires interpretation in the context of the overall clinical phenotype [8].

Anti‐synthetase syndrome (ASS) was specifically considered due to the presence of anti‐PL‐7 antibodies. ASS is typically characterized by interstitial lung disease, myositis, arthritis, Raynaud′s phenomenon, and mechanic′s hands [9] In this case; however, no interstitial lung disease was identified on CT, and there were no clinical or biochemical signs of myositis, nor other defining extramuscular features of ASS. In addition, the anti‐PL‐7 reactivity was weakly positive, limiting its diagnostic specificity in isolation.

Although myocarditis may be more commonly associated with inflammatory myopathies, the overall clinical phenotype in this patient—characterized by longstanding digital ischemia with ulceration, progressive dysphagia, and severe cachexia—is more consistent with dcSSc. Cardiac involvement in dcSSc may include inflammatory myocardial changes in addition to fibrotic remodeling, particularly in advanced disease.

Taken together, the findings support systemic sclerosis as the primary unifying diagnosis. However, an overlap syndrome cannot be definitively excluded, and the serologic findings are interpreted as supportive but not disease‐defining.

Cardiac involvement is a poor prognostic marker, often resulting from irreversible myocardial changes in the advanced stages of dcSSc [2]. The pathophysiology of primary cardiac involvement in systemic sclerosis is believed to be multifactorial. Endothelial injury and microvascular dysfunction represent early disease processes and may lead to recurrent episodes of myocardial ischemia‐reperfusion injury despite the absence of epicardial coronary artery disease. Over time, chronic hypoperfusion promotes myocardial inflammation, myocyte loss, and replacement fibrosis. This diffuse fibrotic process can affect both ventricles and the cardiac conduction system, resulting in systolic dysfunction, arrhythmias, conduction abnormalities, and HF. Cardiac magnetic resonance studies have demonstrated that myocardial edema and fibrosis are common findings in patients with clinically significant cardiac involvement and are associated with adverse outcomes. The severe biventricular dysfunction, atrial fibrillation, ventricular arrhythmias, and low cardiac output observed in the present patient are consistent with advanced myocardial involvement in systemic sclerosis [2, 4]. Once clinically overt HF develops, prognosis is poor, particularly in patients with concomitant renal dysfunction or multiorgan involvement, as observed in the present case [3]. Although myocarditis was suspected based on cardiac MRI findings, it is likely that chronic microvascular injury and progressive myocardial fibrosis had been present for several years before presentation, explaining the advanced degree of ventricular dysfunction at diagnosis. dcSSc can involve all cardiac structures, with complications classified as either primary, resulting from myocardial fibrosis, or secondary, due to pulmonary arterial hypertension, interstitial lung disease, or scleroderma renal crisis [2]. In this case, MRI findings of myocardial edema were consistent with myocarditis related to dcSSc, rather than secondary causes. We treated the myocarditis according to present guidelines [10].

Although stabilizing with diuretics and dobutamine improved cardiac function, treatment options remain limited for cardiac complications in dcSSc [2]. Though cardiac disease is a leading cause of mortality in dcSSc [11], the immediate cause of death in this case was gastrointestinal perforation due to stress ulcerations (and high‐dose corticosteroids) and sepsis, exacerbated by the patient′s malnutrition and immunocompromised state.

Furthermore, whether the treatment for dcSSc ultimately harmed the patient or contributed to an improvement in the condition is hard to evaluate since the outcome was death. However, all treatments initiated were fully in accordance with established clinical guidelines [12].

Several case reports have described myocarditis and acute HF as manifestations of systemic sclerosis. Ramalho et al. reported autoimmune myocarditis with severe left ventricular dysfunction occurring early in the disease course, with near‐complete recovery following immunosuppressive treatment and guideline‐directed HF therapy [13]. Similarly, isolated cases of scleroderma myocarditis presenting with acute congestive HF have been reported, highlighting myocardial inflammation as a potentially reversible cause of cardiac dysfunction when recognized early [14].

Compared with these previously published cases, our patient presented with exceptionally advanced disease characterized by severe biventricular failure (LVEF 10%), markedly reduced cardiac index (1.1 L/min/m^2^), ventricular arrhythmias, dialysis‐dependent renal failure, severe malnutrition, and ultimately a fatal outcome. Furthermore, significant interstitial lung disease and pulmonary hypertension were absent, suggesting primary myocardial involvement as the dominant disease manifestation. This case therefore expands the existing literature by illustrating how delayed recognition of dcSSc may lead to end‐stage cardiac involvement and multiorgan failure before diagnosis is established. This case also highlights the importance of a multidisciplinary approach in managing systemic sclerosis with HF due to its complex, multisystem nature. Cardiac and renal complications demand early detection through specialized monitoring by appropriate specialists. Collaboration between cardiologists, rheumatologists, and nephrologists ensures comprehensive care and handling.

## 5. Conclusion

Primary cardiac involvement as an initial manifestation of SSc is rare and remains a significant source of morbidity and mortality.

**Nomenclature**
CT: Computer tomography
dcSSc: Diffuse cutaneous systemic sclerosis
HF: Heart failure
MRI: Cardiac magnetic resonance imaging

## Funding

No funding was received for this manuscript.

## Disclosure

The authors have nothing to report.

## Consent

The patient allowed personal data processing and informed consent was obtained from the patient included in this case report.

## Conflicts of Interest

The authors declare no conflicts of interest.

## Supporting Information

Additional supporting information can be found online in the Supporting Information section.

## Supporting information


**Supporting Information 1** Video S1: Title: Echocardiographic four‐chamber view at admission. Legend: four‐chamber view with severely decreased left ventricular function (ejection fraction 10%).

**Supporting Information 2** Video S2: Title: Echocardiographic apical long axis view at admission. Legend: Apical long axis view with severely decreased left ventricular function (ejection fraction 10%).

**Supporting Information 3** Video S3: Title: Echocardiographic visualization of tricuspid regurgitation at admission. Legend: Severely decreased left ventricular function (10%) and a moderate to severe tricuspid regurgitation with a dilated right ventricle and a tricuspid annular plane systolic excursion of 11 mm.

**Supporting Information 4** Video S4: Title: Follow‐up echocardiographic four‐chamber view (Day 11) during dobutamine infusion (3 *μ*g/kg/min). Legend: four‐chamber view with increased left ventricular ejection fraction (35%–40%) during Dobutamine infusion. A nonhemodynamic pericardial effusion was observed.

**Supporting Information 5** Video S5: Title: Echocardiographic apical long axis view during dobutamine infusion (3 *μ*g/kg/min). Legend: An increase in left ventricular function (35%–40%) during dobutamine infusion and a circumscript pericardial effusion was observed.

## Data Availability

The data that support the findings of this study are available from the corresponding author upon reasonable request.
